# Predicting hypercapnia and hypoxia by the ventilator's built-in software in children on long-term non-invasive ventilation: A pilot study

**DOI:** 10.3389/fped.2023.1158396

**Published:** 2023-04-24

**Authors:** Xante Mentens, Janne Vanhees, Jolien Paulussen, Sophie Installé, Anse Van Ostaeyen, Kris Ides, Nathalie Jouret, Kim Van Hoorenbeeck, Stijn Verhulst

**Affiliations:** ^1^ Faculty of Medicine and Health Sciences, University of Antwerp, Wilrijk, Belgium; ^2^ Department of Pediatrics, Antwerp University Hospital, Edegem, Belgium

**Keywords:** child, telemonitoring, follow up, non-invasive ventilation, built-in software, hypercapnia, desaturation

## Abstract

**Introduction:**

Follow-up of children on long-term non-invasive ventilation (NIV) could be improved by telemonitoring, using the ventilator's built-in software (BIS) parameters as alternative for in-hospital sleep studies to reduce costs, enhance patient independence and contribute to early detection of infections. This pilot study investigated whether analysis of BIS parameters can predict abnormal nocturnal transcutaneous CO2 (TcCO2) and saturation (SpO2) measurements in children on long-term NIV.

**Methods:**

Children on long-term NIV in follow-up at the Antwerp University Hospital were retrospectively included. Nocturnal TcCO2 and SpO2 measurements were collected together with BIS parameters at three different time points: the night of the sleep study (BIS_1_), mean values from 48 h (BIS_2_) and 72 h (BIS_3_) before the sleep study. Predictions were calculated for following outcome measures: % recording time TcCO2 > 46.9 mmHg (%RT TcCO2; abnormal if ≥2%), recording time SpO2 < 93% (RT SpO2; abnormal if >1 h), abnormal TcCO2 or SpO2, mean TcCO2, mean SpO2.

**Results:**

69 patients were included. %RT TcCO2 was separately predicted by reached tidal volume_2_ [OR 0.97 (0.93; 1.00); *p* = 0.051; AUC = 30%] and reached IPAP_1_ [OR 1.05 (1.00; 1.10); *p* = 0.050; AUC = 66%]. Leak_1_ predicted RT SpO2 [OR 1.21 (1.02; 1.43); *p* = 0.025; AUC = 84%]. Mean TcCO2 correlated with reached tidal volume_2_ (*R*^2^ 0.10, *p* = 0.033).

**Discussion:**

Certain BIS parameters can predict nocturnal hypercapnia and desaturation in children on long-term NIV. Future studies with larger sample sizes are warranted to further investigate the predictive value of the identified BIS parameters.

## 1. Introduction

The population of children receiving long-term non-invasive home ventilation is rapidly expanding (1). Indications for the use of long-term non-invasive ventilation (NIV) include a wide range of disorders resulting in respiratory insufficiency, such as neuromuscular, pulmonary or central nervous system diseases and obstructive sleep apnea syndrome (2). Follow-up of children receiving NIV at home should occur regularly to check tolerance and efficacy of the treatment (3). However, no validated guidelines are available for monitoring of these children. Most institutions perform a sleep study at least annually during overnight hospitalization, by monitoring transcutaneous CO2 (TcCO2) and oxygen saturation (SpO2). Besides hospitalizations, follow-up may consist of home visits and outpatient clinic visits. Moreover, improvements of the built-in software (BIS) of ventilator devices allow for data obtained from these devices to be used in clinical practice, often accessible via telemonitoring (3).

Trucco et al. investigated to what extent telemonitoring (TM) would improve home management of children using home mechanical ventilation. They concluded that the number of hospital admissions due to respiratory infections was significantly reduced in TM patients and duration of hospital admission was significantly shorter (4). Zhou et al. found a telemonitoring system for children using long-term NIV to be reliable and safe, with high caregiver satisfaction and overall reduction of costs (5). If physicians can rely on correct data from BIS, exacerbations or other problems could largely be solved at home reducing the time between the occurrence and solving of problems (4, 5). TM could also be useful in optimizing the settings of NIV. In contrast to a single overnight sleep study, long-term data from TM gives the physician more information to adjust the settings according to changes in disease state (6). TM and home management could improve patients' independence, self-awareness and understanding about their condition (5, 6). However, there is little evidence regarding strengths and disadvantages of using BIS in pediatrics (7). Onofri et al. investigated the validity of BIS in the follow-up of children on NIV by analyzing the ability of BIS to identify residual sleep events. The apnea-hypopnea index (AHI) scores based on the BIS were positively correlated, although weakly, with the AHI scores of polygraphy (8). Khirani et al. concluded that manual analysis of the BIS tracing of CPAP devices, with addition of a SpO2 signal markedly reduced the differences of AHI values between the CPAP and polygraphy scoring (9).

Parameters obtained from the BIS are calculated using algorithms. However, it is uncertain whether these calculated parameters correspond with measurements during sleep. To our knowledge, a direct correlation between the analysis of BIS and the results of nocturnal TcCO2 and SpO2 measurements has not been performed yet. If this study could demonstrate such direct correlation and establish a predictive model for abnormal nocturnal TcCO2 and SpO2 values, (tele)monitoring via parameters from BIS could represent a feasible alternative for in-hospital sleep studies. Thus, the aim of this study is to determine the diagnostic accuracy of BIS parameters for detecting an abnormal nocturnal TcCO2 and SpO2 value in children and adolescents on long-term non-invasive ventilation.

## 2. Methods

### 2.1. Study design and population

This retrospective study included all eligible patients from the Antwerp University Hospital who received home non-invasive ventilation between October 2017 and September 2021. Inclusion criteria were age between 0 and 18 years old, use of long-term non-invasive bilevel ventilation, stable condition during nocturnal TcCO2 and SpO2 monitoring, compliance with NIV (using it more than 6 h per night). Exclusion criteria were exclusive use of CPAP and acute infection at the time of nocturnal TcCO2 and SpO2 measurements.

The study was approved by the Ethical Committee of the Antwerp University Hospital. Informed consent was waived due to the retrospective nature of the study. To ensure patient privacy, data was coded for processing and preserved in a secured database at the Antwerp University Hospital.

### 2.2. Data collection

The following baseline characteristics were collected for each patient: age, gender and disorder from which they are suffering. The disorders were classified in the following categories: increase in respiratory load, decrease in performance of respiratory muscles and dysfunction of central drive (1).

TcCO2 and SpO2 data was collected by an in-hospital sleep study comprised of overnight monitoring of the oxygen saturation and transcutaneous capnography, by standard pediatric recording and scoring techniques. Transcutaneous CO2 is measured with a pH-electrode, which causes a local hyperemia of the skin up to 42°C to increase local blood flow. The CO2 sensed by the electrode corresponds to the PaCO2. Oxygen saturation is monitored by pulse oximetry, which detects oxygen saturation by absorption of light of two different wave lengths, affected by the oxygen saturation of arterial blood. Measurements were performed using the SenTec Digital Monitoring System according to the manufacturer's instructions and analyzed using the V-STATS™ software. Measurements of interest regarding TcCO2 are mean TcCO2 and % of recording time with TcCO2 > 46.9 mmHg. Similarly, for SpO2, these measurements are mean SpO2 and amount of recording time with SpO2 < 93%.

The Philips Respironics Trilogy 100 ventilator was used in all patients with ventilation modes S/T with AVAPS, S/T without AVAPS and PC-SIMV. Different sizes of mask interfaces were used depending on face shape and patient comfort, with most children using a nasal mask. From the analysis of the built-in software of the ventilator, the following data were collected: exhaled tidal volume (TV) (ml), reached TV (% of Target TV), mean inspiratory positive airway pressure (IPAP) (cmH2O), reached IPAP (% of set IPAP), mean expiratory positive airway pressure (EPAP) (cmH2O), reached EPAP (% of set EPAP), mean respiratory rate (RR) (bpm), reached RR (% of back-up frequency), triggering (%), leak (L/min). These values were calculated at different points in time: during the night of the TcCO2 and SpO2 measurement during the sleep study (BIS_1_), the average values of 48 h before the sleep study (BIS_2_) and the average values of 72 h before the sleep study (BIS_3_). The analysis of these variables is carried out continuously by the built-in software if the patient uses the ventilator.

### 2.3. Statistical analyses

Regarding the TcCO2 and SpO2 measurements during sleep, continuous data were collected and used as dependent variables. The primary outcome variable was the percentage of recording time with TcCO2 > 46.9 mmHg (%RT TcCO2). This cutoff value of 46.9 mmHg was chosen according to the technical settings of the SenTec Digital Monitoring System. For the statistical analyses, two groups were created based on whether or not this percentage is equal to or above 2% (10). As such, %RT TcCO2 was considered a dichotomous variable (abnormal if %RT TcCO2 ≥ 2%). Regarding secondary outcomes, the amount of recording time with SpO2 < 93% (RT SpO2) was also considered as dichotomous. This variable was abnormal when the amount of recording time with SpO2 < 93% exceeds one hour^1^. The two aforementioned parameters were combined to form the third outcome variable %RT TcCO2-RT SpO2. This dichotomous variable was considered abnormal if either %RT TcCO2 or RT SpO2 were abnormal, according to the descriptions above. The last two outcomes were mean TcCO2 and mean SpO2. These variables were regarded as dichotomous. Groups were created with cutoff values 46.9 mmHg for mean TcCO2 (abnormal if >46.9 mmHg) and 93% for mean SpO2 (abnormal if <93%). However, these outcome variables were also analyzed as continuous variables. The BIS parameters mentioned in “data collection” were considered independent variables.

Statistical analyses were carried out using IBM SPSS28. *P* < 0.05 was considered as statistically significant. Missing data were excluded case by case from the analyses. Comparison of baseline characteristics between groups was conducted using an independent two-sample t-test, Chi Square test or Fisher exact test when appropriate. To compare the variables from the BIS analysis between groups, an independent two sample t-test or Mann-Whitney U-test was used. Next, Spearman or Pearson correlations were calculated between baseline characteristics and the outcome variable. The same correlation analyses were performed between BIS parameters and the outcome variable. All baseline characteristics and BIS parameters identified as significantly different between groups or significantly correlating, were used in a logistic regression analysis. This way, a model was created to predict an abnormal outcome variable. Complementary to the logistic regression analysis, ROC curves were generated and area under the curve (AUC) was calculated to determine the predictive magnitude of the model.

The secondary outcomes mean TcCO2 and mean SpO2 were also analyzed as continuous variables. Correlation analyses with Spearman or Pearson were performed between baseline characteristics and the continuous variable and between BIS parameters and the continuous variable. The baseline characteristics and BIS parameters identified as significantly correlating, were used in a linear regression analysis, if assumptions for linear regression analysis were met. Thus, for mean TcCO2 and mean SpO2 both a linear and a logistic prediction model were generated.

## 3. Results

### 3.1. Study population

A total of 69 patients were included in the database. The median age of the patients were 36 months (minimum of 2 weeks; maximum of 18 years). 38 of the included patients were boys (55%) and 31 of the included patients were girls (45%). There were 30 patients (43%) who suffered from a disorder with an increase in respiratory load (most frequent encountered disorders were obstructive sleep apnea syndrome, bronchiolitis obliterans and Pierre Robin syndrome). 28 patients (41%) suffered from a disorder with decreased performance of respiratory muscles (most frequent encountered disorders were spinal muscular atrophy, myopathy and muscle dystrophy). 11 patients (16%) suffered from a disorder with dysfunction of central drive (most frequent encountered disorder was congenital central hypoventilation syndrome). Additional study population characteristics regarding ventilation use (mode, frequency and timing) can be found in Table 1 of the Supplementary Material.

There were no missing values for baseline characteristics. From a total of 69 measurements nine values (13%) were missing for TV, IPAP, reached IPAP, EPAP, reached EPAP, mean RR, reached RR, triggering and leak during the night of the sleep study and 48 h before the sleep study. 13 out of 69 values (19%) were missing for the same BIS parameters 72 h before the sleep study. 21 values (30%) were missing for reached TV_1_, 23 values (33%) were missing for reached TV_2_ and 27 values (39%) were missing for reached TV_3_. Missing values for outcome variables will be mentioned for each variable further in this section.

### 3.2. Primary outcome

A total of 66 measurements was used in the analyses for the primary outcome %RT TcCO2 with 45 measurements being normal and 21 measurements being abnormal. Three measurements were excluded because of a missing value for %RT TcCO2. There were no significant differences between groups for all three baseline characteristics. Two BIS parameters were significantly different between groups; reached TV_2_ (*p* = 0.027) and reached IPAP_1_ (*p* = 0.043). No other differences were observed. No baseline characteristics were significantly correlating with %RT TcCO2. The two BIS parameters reached TV_2_ (*p* = 0.026) and reached IPAP_1_ (*p* = 0.043) correlated significantly with %RT TcCO2 (Table 1).

**Table 1:** Statistically significant results for comparison of baseline characteristics and BIS parameters between groups and correlation analyses for primary and secondary outcomes.

	Comparison between groups			Correlation analysis	
	Normal group	Abnormal group	*p*-value	Correlation coefficient	*p*-value
*%RT TcCO2*					
Reached TV_2_ (% of target TV)	111.18 (127.18)	89.26 (76.39)	*p* = 0.027*	ρ = −0.33	*p* = 0.026*
Reached IPAP_1_ (% of set IPAP)	80.96 ± 12.65	88.28 ± 11.77	*p* = 0.043*	r = 0.27	*p* = 0.043*
*RT SpO2*					
Reached EPAP_3_ (% of set EPAP)	99.75 (33.53)	99.50 (1.03)	*p* = 0.046*	ρ = −0.27	*p* = 0.047*
Leak_1_ (L/min)	36.91 (31.98)	48.40 (57.52)	*p* = 0.004*	ρ = 0.36	*p* = 0.005*
Leak_2_ (L/min)	35.35 (31.81)	44.04 (117.90)	*p* = 0.049*	ρ = 0.26	*p* = 0.048*
*Mean SpO2 (logistic)*					
Reached IPAP_1_ (% of set IPAP)	82.81 ± 12.73	93.85 ± 2.58	*p *< 0.001*		
*Mean TcCO2 (linear)*					
TV_1_ (ml)				ρ = −0.26	*p* = 0.045*
Reached TV_2_ (% of target TV)				ρ = −0.31	*p* = 0.038*

1 = value from the night of the TcCO2 and SpO2 measurement; 2 = value from 48h before the overnight measurement; 3 = value from 72 h before the overnight measurement. *ρ* = Spearman correlation coefficient; *r* = Pearson correlation coefficient. Normally distributed data are presented as mean ± standard deviation (SD) and skewed data as median (range).

* Statistical significance is indicated by an asterisk.

Logistic regression analysis was carried out for %RT TcCO2 with reached TV_2_ and reached IPAP_1_ as determinants, as well as the interaction term reached TV_2_*reached IPAP_1_ because of the strong correlation between the two BIS parameters (ρ = −0.56; *p* < 0.001). A prediction model with both reached TV_2_ and reached IPAP_1_ was not significant, neither was the interaction term reached TV_2_*reached IPAP_1_. To exclude effects of multicollinearity, both BIS parameters were also used in separate logistic regression analyses. A prediction model with reached TV_2_ as sole determinant was nearly significant [*p* = 0.051; OR 0.97 (0.93; 1.00); AUC = 30%]. A prediction model with reached IPAP_1_ as sole determinant was also close to significance [*p* = 0.050; OR 1.05 (1.00; 1.10); AUC = 66%].

### 3.3. Secondary outcomes

**Figure 1:**
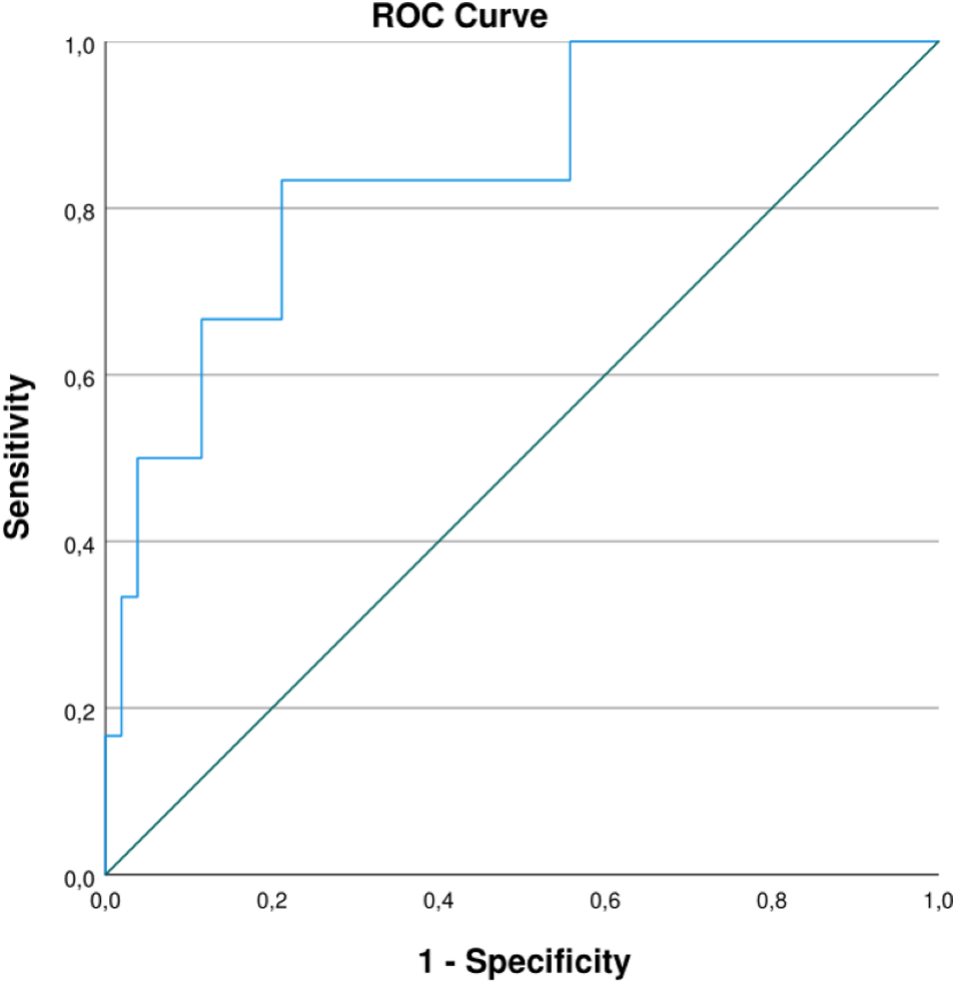
ROC curve of logistic prediction model for RT SpO2 by leak_1_. AUC of 84%. Sensitivity of 83.3% and specificity of 78.8% with a cutoff value of 42.16 L/min.

The first secondary outcome was the dichotomous variable RT SpO2. A total of 67 measurements was used in the analyses with 60 measurements being normal and 7 measurements being abnormal. Two measurements were excluded because of a missing value for RT SpO2. For the baseline characteristics there were no significant differences between groups, nor statistically significant correlations with RT SpO2. Three BIS parameters were significantly different between groups, i.e., reached EPAP_3_ (*p* = 0.046), leak_1_ (*p* = 0.004) and leak_2_ (*p* = 0.049). The same three BIS parameters were also significantly correlated with RT SpO2 in Spearman correlation analyses (Table 1). Logistic regression analysis was carried out for RT SpO2 with these three parameters. The prediction model with leak_1_ as sole determinant was statistically significant [*p* = 0.025; OR 1.21 (1.02; 1.43)] (Table 2). The ROC curve of this prediction model is shown in Figure 1 with an AUC of 84%. When looking to the test properties of leak_1_ as predictive test for desaturation, a cutoff value of 42.16 L/min would result in a sensitivity of 83.3% and a specificity of 78.8%.

**Table 2:** Logistic and linear regression analysis for %RT TcCO2, RT SpO2 and mean TcCO2 by BIS parameters.

	Regression coefficient (95% CI if linear)	*p*-value	Odds Ratio (95% CI)	*R*²
*% RT TcCO2 (logistic)*				
Reached TV_2_	−0.04	*p* = 0.051	0.97 (0.93; 1.00)	
Constant	2.90	*p* = 0.105		
*% RT TcCO2 (logistic)*				
Reached IPAP_1_	−0.05	*p* = 0.050	1.05 (1.00; 1.10)	
Constant	−4.87	*p* = 0.023*		
*RT SaO2 (logistic)*				
Leak_1_	0.189	*p* = 0.025*	1.21 (1.02; 1.43)	
Constant	−10.16	*p* = 0.007*		
*Mean TcCO2 (linear)*				
Reached TV_2_	−0.11 (−0.21; −0.01)	*p* = 0.033*		0.10
Constant	49.35 (38.93; 59.78)	*p* < 0.001*		

1 = value from the night of the TcCO2 and SpO2 measurement; 2 = value from 48 h before the overnight measurement; 3 = value from 72 h before the overnight measurement. CI, confidence interval.

* Statistical significance is indicated by an asterisk.

Another dichotomous secondary outcome variable was %RT TcCO2-RT SpO2. A total of 69 measurements was used in the analyses with no missing values for the outcome variable. Two groups were created with 45 measurements in the normal group and 24 in the abnormal group. For both baseline characteristics and BIS parameters there were no significant differences between groups, nor were there significantly correlating parameters with the outcome variable. Since no parameters could be selected from previous analyses, no logistic regression analysis was carried out.

The secondary outcome variable mean SpO2 was analyzed as dichotomous and continuous variable, using a total of 69 measurements. For the dichotomous analyses, two groups were created with the normal group containing 64 measurements and the abnormal group containing five measurements. For the baseline characteristics there were no significant differences between groups, nor statistically significant correlations with mean SpO2 as dichotomous variable. The BIS parameter reached IPAP_1_ was significantly different between groups (*p* < 0.001), however it did not correlate significantly with the outcome variable (Table 1). A logistic prediction model for mean SpO2 by reached IPAP_1_ was not statistically significant. For mean SpO2 as a continuous variable, there were no significantly correlating baseline characteristics or BIS parameters. Therefore, no linear regression analysis was performed.

The last secondary outcome variable was mean TcCO2, which was also analyzed as dichotomous and continuous variable. A total of 69 measurements was used in the analyses (no missing values for the outcome variable). For the dichotomous analyses, two groups were created with 59 measurements in the normal group and 10 measurements in the abnormal group. For both baseline characteristics and BIS parameters there were no significant differences between groups, nor were there parameters significantly correlating with mean TcCO2 as dichotomous variable. No logistic regression analysis was carried out for mean TcCO2 since no parameters could be selected from previous analyses. Correlation analyses were performed between baseline characteristics and BIS parameters with mean TcCO2 as continuous variable. BIS parameters TV_1_ and reached TV_2_ were significantly correlated with mean TcCO2 (respective *p*-values of 0.045 and 0.038) (Table 1). A linear regression model for mean TcCO2 by reached TV_2_ was generated (*R*^2^ 0.10; *p* = 0.033) (Table 2). Detailed results of the comparison and correlation analyses of all outcome variables can be found in Supplementary Tables 2–6.

## 4. Discussion

This retrospective, pilot study investigated whether BIS parameters of the ventilator could predict abnormal values of nocturnal TcCO2 and SpO2 in children on long-term non-invasive ventilation.

Results indicate that certain BIS parameters, i.e., reached TV, reached IPAP and leak, may be able to predict nocturnal hypercapnia and desaturation. The mean reached TV of 48 h before nocturnal measurements was a significant parameter in a linear regression model for mean TcCO2. For %RT TcCO2 as well, reached TV_2_ appeared to be an important parameter with possible predictive properties, based on the logistic prediction model where there was a trend towards statistical significance. As such, this BIS parameter is clearly a parameter of interest in relation to hypercapnia. Regarding hypercapnia, the reached IPAP during the night of nocturnal measurements also showed potential predictive qualities. Like reached TV_2_, the logistic prediction model for %RT TcCO2 with reached IPAP_1_ demonstrated a trend towards statistical significance. For the prediction of nocturnal desaturation, the BIS parameter leak_1_ seemed to be relevant. Logistic regression analysis resulted in a statistically significant prediction model for RT SpO2 with an AUC of 84%. This shows that leak_1_ possibly has a considerable predictive value with regards to nocturnal desaturation. The cutoff for leak_1_ as a predictive test for desaturation can be set at 42.16 L/min to maximize both the sensitivity (83.3%) and specificity (78.8%) of the test, with emphasis on optimal sensitivity to avoid false negative results. A leak flow of 42.16 L/min seems applicable as cutoff value in clinical practice based on observations by clinical experts in the field.

The identified BIS parameters seem to have a predictive value at only one time point, but not at the other time points. In literature, no explanation is offered for this finding, although it does not seem entirely illogical. If for example a child becomes ill due to a respiratory infection, TcCO2 and SpO2 could become abnormal during a certain night. It is expected that there would already be changes in the respiratory parameters ahead of that specific night. For this reason, this study has not only investigated BIS parameters during the night of the sleep study but also the period of 48 h and 72 h before. Besides, as an illness usually develops gradually over a period of days/hours, it is hypothesized that changes in respiratory parameters—and thus BIS parameters—would also occur gradually until a new homeostatic equilibrium is reached. This dynamic nature of illness could possibly explain in part why certain BIS parameters are for example only significant in the 48 h before the sleep study.

There is a considerable amount of literature available about monitoring of NIV and the use of ventilator built-in software. Built-in software (BIS) offers a lot of new data that is intuitively useful and most parameters appear to be reliable in monitoring of NIV (6, 11).

Several clinical trials already confirmed the usefulness of BIS parameters in the prediction of outcomes such as AHI in patients with obesity-hypoventilation syndrome and exacerbation in patients with chronic obstructive pulmonary disease (12, 13). Currently, no validated guidelines exist for monitoring of NIV using BIS parameters, although certain parameters emerge from literature as useful, e.g., leak and exhaled tidal volume (14). This pilot study identified three such BIS parameters: reached tidal volume, reached IPAP and leak. Previous studies confirmed the clinical relation between nocturnal hypercapnia and TV; low tidal volumes can lead to nocturnal hypoventilation with hypercapnia (15, 16). Because of this, monitoring of TV seems to be a key point in the evaluation of efficacy of NIV (17). Nevertheless, estimations of TV by BIS are often inaccurate and reliability greatly depends on the type of ventilator, pressure settings and the amount of leak (6, 11, 18). For the BIS parameter IPAP as well, a link with nocturnal hypercapnia can be derived from literature. The ventilator setting for IPAP is usually titrated according to achieve adequate CO2 removal (2). When hypoventilation with hypercapnia is observed, it is advised to increase the value of IPAP administered to the patient (16, 18). The BIS parameter leak is also much discussed in literature. Unintentional leaks can lead to patient-ventilator asynchrony and nocturnal hypoventilation with associated desaturation, which can be overcome by adjusting the ventilator interface (11, 18). Estimations of leak differ greatly according to the ventilator device tested, although several studies describe these estimations by BIS as reliable nonetheless (6, 11, 16, 19). Furthermore, leak has a large impact on other estimations by BIS, such as TV and percentage of cycles triggered (18).

In contrast to the extensive debate in literature about the value and reliability of individual BIS parameters, unanimity exists about the presence of large discrepancies between different software systems (16). It would be convenient to reach a consensus between manufacturers on measuring and reporting data of BIS with homogenized algorithms (16, 20). If this is not possible, conclusions based on information provided by BIS should only be considered as indicative because the lack of inter-algorithm comparability.

This study has several strengths. To our knowledge, this was the first pediatric study to try and find a prediction model for TcCO2 and SpO2 based on the ventilator's BIS parameters. an extensive number of variables was collected, which enhanced the chances of finding a possible prediction model. With future validation of a prediction model, the use of telemonitoring could be expanded which brings about several advantages. Trucco et al. showed that telemonitoring was effective in reducing hospital admissions due to respiratory infections (4). Zhou et al. confirmed reliability, safety, cost-effectiveness and improved caregiver satisfaction of a telemonitoring system for children using long-term NIV (5). TM increases patients’ independence by augmenting their self-awareness and understanding about the condition (6). With telemonitoring using BIS as an equally effective alternative, children would not have to visit the hospital that often and in-hospital sleep studies can be preserved for more complex cases (6, 21). Georges et al. also showed that analysis of BIS parameters in combination with CO2 measurements is the best way to monitor NIV compared to several combinations of arterial blood gas, oximetry, TcCO2 and BIS parameters (21). As an additional strength, the children included in the study only underwent examinations that were part of standard care, without any additional burden or risks by extra interventions. This also ensures that the results of this study are based on the realistic clinical condition of the patients as encountered in daily practice. By only using data from examinations part of traditional follow-up, the conclusions from this study are directly applicable to real world clinical care without major modifications to current practice.

Besides the strengths in this study, there are also some limitations. First of all, the power of the results in this study was limited by the small sample size. the group counts of normal and abnormal groups were not equally distributed for certain dichotomous outcome variables. It was also observed that the constructed regression models were not that well-fitting, with exception of the model for RT SpO2 (AUC of 84%). This could be partly related to the small sample size. Secondly, given the large number of parameters used in the study, the multiple comparisons problem arose. As no adjustments were made for multiple testing, it is important to keep in mind that false positive results may occur. Furthermore, since this study has a retrospective design, missing data could not be prevented. The number of missing values for the outcome measures was limited, but for the different BIS parameters, the percentage of missing data ranged from 13% to 39% of the total values. In this study only a single type of ventilator was used (Philips Respironics Trilogy 100 ventilator) and results could possibly be different when using another type of ventilator. Different size of mask interfaces were used by patients included in this study, making the cutoff value for leak less universally applicable. Additionally, the cutoff values used in this study do not correspond to international definitions of hypercapnia and hypoxemia (10). Lastly, there are no known prediction models for TcCO2 or SpO2 at the current moment. Therefore, the results from this study could not be compared to previous studies.

In conclusion, this pilot study supports the hypothesis that abnormal nocturnal TcCO2 and SpO2 measurements could be predicted by certain parameters obtained from analysis of the ventilator's built-in software in children using long-term non-invasive ventilation. The BIS parameters of interest identified in this study are reached IPAP and leak during the night of nocturnal measurements (reached IPAP_1_, leak_1_) and mean reached tidal volume from 48 h before nocturnal measurements (reached TV_2_). As this study is a pilot study, it is warranted to further investigate these BIS parameters and validate the possible prediction models. A prospective, multicenter clinical trial with a larger sample size and use of additional ventilator types could be conducted in further exploration of this topic and the possibilities of telemonitoring in children on long-term non-invasive ventilation.

## Data Availability

The raw data supporting the conclusions of this article will be made available by the authors, without undue reservation.

## Footnotes

^1^This definition of nocturnal hypoxemia was based on the Belgian national reimbursement criteria for oxygen therapy in children.
