# Supplementary material for: Predicting hypercapnia and hypoxia by the ventilator's built-in software in children on long-term non-invasive ventilation: A pilot study
Source: Front Pediatr. 2023 Apr 24;11:1158396. doi: 10.3389/fped.2023.1158396 (PMC10166201; doi:10.3389/fped.2023.1158396)
Supplement: Supplementary file 1 [file Datasheet1.pdf]

Supplementary Material

Predicting hypercapnia and hypoxia by the ventilator's built-in software in children on chronic non-invasive ventilation: a pilot study.

Xante Mentens[1†*], Janne Vanhees[1†], Jolien Paulussen[1†], Sophie Installé[2], Anse Van Ostaeyen[2], Kris Ides[2], Nathalie Jouret[2], Kim Van Hoorenbeeck[2], Stijn Verhulst[1,2]

[1]Faculty of Medicine and Health Sciences, University of Antwerp, Wilrijk, Belgium.

[2]Department of Pediatrics, Antwerp University Hospital, Edegem, Belgium.

†These authors contributed equally to this work and share first authorship.

*** Correspondence:**
Xante Mentens
xante.mentens@hotmail.com

1 Supplementary Figures and Tables

Supplementary table 1: Characteristics of the study population characteristics and BIS
S/T = Spontaneous/Timed, AVAPS = Average volume-assured pressure support, PC-SIMV = Pressure control synchronized intermittent mandatory ventilation

**Study population**		
**Age (median, range)**		36 months (2 weeks; 18 years)
**Sex (% male)**		55
**Disorder (%)**	Increase in respirator load	43
	Decreased performance of respiratory muscles	41
	Decreased performance of respiratory muscles	16
**Ventilation mode (%)**	S/T with AVAPS	78
	S/T without AVAPS	16
	PC-SIMV	6
**Frequency of ventilator use (%)**	Intermittent	19
	Daily	81
**Timing of ventilator use (%)**	Nighttime	76
	Daytime	4
	Continuous	20

Supplementary table 2: Comparison of baseline characteristics and BIS parameters between groups and correlation analyses for %RT $TcCO_2$.

1 = value from the night of the $TcCO_2$ and SpO_2 measurement; 2 = value from 48h before the overnight measurement; 3 = value from 72 hours before the overnight measurement. ρ = Spearman correlation coefficient; r = Pearson correlation coefficient. Normally distributed data are presented as mean ± standard deviation (SD) and skewed data as median (range). Statistical significance is indicated by an asterisk.

**BIS parameters**		**Comparison between groups**			**Correlation analysis**	
		Normal %RT TcCO2 (n = 45)	Abnormal %RT TcCO2 (n = 21)	p-value	Correlation coefficient	p-value
TV (ml)	TV_1	120.87 (302.73)	134.10 (401.43)	p = 0.77	ρ = -0.04	p = 0.77
	TV_2	111.42 (337.15)	117.11 (286.22)	p = 0.81	ρ = -0.03	p = 0.81
	TV_3	117.54 (331.63)	108.00 (286.44)	p = 0.85	ρ = -0.03	p = 0.86
Reached TV (% target TV)	Reached TV_1	102.45 (190.52)	93.30 (106.48)	p = 0.12	ρ = -0.24	p = 0.12
	Reached TV_2	111.18 (127.18)	89.26 (76.39)	p = 0.03*	ρ = -0.33	p = 0.03*
	Reached TV_3	100.27 (125.67)	88.15 (59.80)	p = 0.10	ρ = -0.26	p = 0.10
IPAP (cmH2O)	$IPAP_1$	16.42 ± 3.70	16.97 ± 4.92	p = 0.64	r = 0.06	p = 0.64
	$IPAP_2$	16.39 ± 3.40	16.40 ± 4.06	p = 0.99	r = 0.001	p = 0.99
	$IPAP_3$	16.34 ± 3.53	16.54 ± 4.33	p = 0.85	r = 0.03	p = 0.85
Reached IPAP (% set IPAP)	Reached $IPAP_1$	80.96 ± 12.65	88.28 ± 11.77	p = 0.04*	r = 0.27	p = 0.04*
	Reached $IPAP_2$	82.26 ± 12.15	84.90 ± 9.76	p = 0.41	r = 0.11	p = 0.41
	Reached $IPAP_3$	82.72 ± 12.18	85.34 ± 9.76	p = 0.43	r = 0.11	p = 0.43
EPAP (cmH2O)	$EPAP_1$	4.99 (6.02)	5.50 (4.01)	p = 0.28	ρ = 0.15	p = 0.28
	$EPAP_2$	4.98 (6.00)	5.51 (4.00)	p = 0.30	ρ = 0.14	p = 0.30
	$EPAP_3$	4.99 (5.99)	5.98 (3.99)	p = 0.20	ρ = 0.18	p = 0.20
Reached EPAP (% set EPAP)	Reached $EPAP_1$	99.67 (26.90)	99.71 (56.17)	p = 0.82	ρ = -0.03	p = 0.82
	Reached $EPAP_2$	99.75 (25.80)	99.71 (56.90)	p = 0.53	ρ = -0.08	p = 0.54
	Reached $EPAP_3$	99.72 (25.85)	99.75 (41.47)	p = 0.59	ρ = 0.07	p = 0.60
Mean RR (bpm)	Mean RR_1	23.76 (31.75)	20.36 (31.08)	p = 0.38	ρ = -0.12	p = 0.39
	Mean RR_2	24.44 (36.19)	20.65 (31.67)	p = 0.53	ρ = -0.08	p = 0.53
	Mean RR_3	23.83 (36.42)	20.87 (33.20)	p = 0.55	ρ = -0.08	p = 0.55
Reached RR (% back-up frequency)	Reached RR_1	112.90 (115.08)	112.49 (62.35)	p = 0.88	ρ = 0.02	p = 0.88
	Reached RR_2	110.13 (85.61)	112.02 (44.21)	p = 0.78	ρ = 0.04	p = 0.78
	Reached RR_3	111.95 (81.55)	113.06 (42.93)	p = 0.93	ρ = 0.01	p = 0.93
Triggering (%)	$Triggering_1$	48.53 ± 23.57	46.17 ± 21.11	p = 0.72	r = -0.05	p = 0.72
	$Triggering_2$	43.93 ± 23.26	45.60 ± 19.58	p = 0.79	r = 0.04	p = 0.79
	$Triggering_3$	45.08 ± 23.64	45.13 ± 18.31	p = 0.99	r = 0.001	p = 0.99
Leakage (L/min)	$Leakage_1$	37.42 (30.61)	38.40 (72.37)	p = 0.54	ρ = 0.08	p = 0.54
	$Leakage_2$	34.44 (31.81)	36.46 (127.29)	p = 0.48	ρ = 0.09	p = 0.49
	$Leakage_3$	33.39 (43.45)	36.46 (123.97)	p = 0.39	ρ = 0.12	p = 0.39

Supplementary table 3: Comparison of baseline characteristics and BIS parameters between groups and correlation analyses for RT SpO2.

1 = value from the night of the TcCO2 and SpO2 measurement; 2 = value from 48h before the overnight measurement; 3 = value from 72 hours before the overnight measurement. ρ = Spearman correlation coefficient; r = Pearson correlation coefficient. Normally distributed data are presented as mean ± standard deviation (SD) and skewed data as median (range). Statistical significance is indicated by an asterisk.

**BIS parameters**		**Comparison between groups**			**Correlation analysis**	
		Normal RT SpO2 (n = 60)	Abnormal RT SpO2 (n = 7)	p-value	Correlation coefficient	p-value
TV (ml)	TV_1	122.33 (400.64)	134.60 (251.69)	p = 0.56	ρ = 0.08	p = 0.55
	TV_2	111.42 (355.95)	187.24 (243.93)	p = 0.30	ρ = 0.14	p = 0.30
	TV_3	117.54 (353.05)	184.51 (244.27)	p = 0.43	ρ = 0.11	p = 0.42
Reached TV (% target TV)	Reached TV_1	97.65 (190.52)	100.27 (70.51)	p = 0.61	ρ = -0.08	p = 0.59
	Reached TV_2	98.55 (127.18)	96.20 (46.29)	p = 0.37	ρ = -0.14	p = 0.36
	Reached TV_3	100.26 (125.67)	98.67 (45.11)	p = 0.36	ρ = -0.15	p = 0.35
IPAP (cmH2O)	$IPAP_1$	16.62 ± 4.43	17.66 ± 2.85	p = 0.58	*r* = 0.07	p = 0.58
	$IPAP_2$	16.68 ± 3.92	16.48 ± 2.33	p = 0.90	*r* = -0.02	p = 0.90
	$IPAP_3$	16.68 ± 4.02	16.80 ± 2.56	p = 0.95	*r* = 0.01	p = 0.95
Reached IPAP (% set IPAP)	Reached $IPAP_1$	83.76 ± 12.87	81.31 ± 10.94	p = 0.66	*r* = -0.06	p = 0.66
	Reached $IPAP_2$	83.81 ± 11.55	78.03 ± 9.45	p = 0.21	*r* = -0.17	p = 0.21
	Reached $IPAP_3$	83.94 ± 11.76	80.18 ± 8.79	p = 0.49	*r* = -0.10	p = 0.49
EPAP (cmH2O)	$EPAP_1$	5.00 (6.01)	6.99 (5.04)	p = 0.09	ρ = 0.23	p = 0.09
	$EPAP_2$	5.00 (5.99)	6.98 (5.06)	p = 0.32	ρ = 0.14	p = 0.31
	$EPAP_3$	5.02 (5.99)	6.98 (5.01)	p = 0.43	ρ = 0.11	p = 0.43
Reached EPAP (% set EPAP)	Reached $EPAP_1$	99.67 (56.17)	99.76 (0.80)	p = 0.51	ρ = 0.09	p = 0.49
	Reached $EPAP_2$	99.75 (56.90)	99.64 (1.25)	p = 0.38	ρ = -0.12	p = 0.36
	Reached $EPAP_3$	99.75 (33.53)	99.50 (1.03)	p = 0.046*	ρ = -0.27	p = 0.047*
Mean RR (bpm)	Mean RR_1	23.17 (32.49)	22.12 (19.96)	p = 0.63	ρ = -0.07	p = 0.61
	Mean RR_2	23.62 (37.18)	18.87 (17.83)	p = 0.27	ρ = -0.15	p = 0.26
	Mean RR_3	22.89 (37.26)	19.30 (15.80)	p = 0.47	ρ = -0.10	p = 0.45
Reached RR (% back-up frequency)	Reached RR_1	112.58 (115.08)	114.80 (16.32)	p = 0.93	ρ = 0.01	p = 0.92
	Reached RR_2	110.18 (85.61)	111.25 (22.13)	p = 0.71	ρ = 0.05	p = 0.69
	Reached RR_3	111.46 (81.55)	116.89 (17.91)	p = 0.26	ρ = 0.16	p = 0.25
Triggering (%)	$Triggering_1$	47.49 ± 23.77	43.71 ± 17.58	p = 0.71	*r* = -0.05	p = 0.71
	$Triggering_2$	42.37 ± 22.60	52.43 ± 18.57	p = 0.27	*r* = 0.15	p = 0.27
	$Triggering_3$	43.01 ± 22.36	53.40 ± 21.18	p = 0.33	*r* = 0.14	p = 0.33
Leakage (L/min)	$Leakage_1$	36.91 (31.98)	48.40 (57.52)	p = 0.01*	ρ = 0.36	p = 0.01*
	$Leakage_2$	35.35 (31.81)	44.04 (117.90)	p = 0.049*	ρ = 0.26	p = 0.048*
	$Leakage_3$	36.46 (43.45)	36.00 (115.88)	p = 0.21	ρ = 0.18	p = 0.20

Supplementary table 4: Comparison of baseline characteristics and BIS parameters between groups and correlation analyses for %RT TcCO2 - RT SpO2.

1 = value from the night of the TcCO2 and SpO2 measurement; 2 = value from 48h before the overnight measurement; 3 = value from 72 hours before the overnight measurement. ρ = Spearman correlation coefficient; r = Pearson correlation coefficient. Normally distributed data are presented as mean ± standard deviation (SD) and skewed data as median (range). Statistical significance is indicated by an asterisk.

BIS parameters		Comparison between groups			Correlation analysis	
		Normal TcCO2-SpO2 (n=45)	Abnormal TcCO-SpO2 (n = 24)	p-value	Correlation coefficient	p-value
TV (ml)	TV_1	123.83 (307.58)	91.47 (401.43)	p = 0.71	ρ = -0.05	p = 0.71
	TV_2	114.87 (337.15)	97.83 (286.22)	p = 0.91	ρ = -0.02	p = 0.91
	TV_3	117.54 (331.63)	111.31 (286.44)	p = 0.96	ρ = 0.01	p = 0.96
Reached TV (% target TV)	Reached TV_1	104.43 (190.52)	95,24 (106.48)	p = 0.16	ρ = -0.21	p = 0.16
	Reached TV_2	101.18 (127.18)	96.20 (76.39)	p = 0.05	ρ = -0.29	p = 0.05
	Reached TV_3	100.50 (125.67)	94.83 (59.80)	p = 0.10	ρ = -0.25	p = 0.10
IPAP (cmH2O)	$IPAP_1$	16.49 ± 4.11	17.05 ± 4.67	p = 0.64	r = 0.06	p = 0.64
	$IPAP_2$	16.68 ± 3.81	16.40 ± 3.81	p = 0.79	r = -0.04	p = 0.79
	$IPAP_3$	16.65 ± 3.88	16.47 ± 4.15	p = 0.87	r = -0.02	p = 0.87
Reached IPAP (% set IPAP)	Reached $IPAP_1$	82.18 ± 12.48	86.29 ± 12.74	p = 0.24	r = 0.16	p = 0.24
	Reached $IPAP_2$	83.41 ± 12.04	83.13 ± 10.54	p = 0.93	r = -0.01	p = 0.93
	Reached $IPAP_3$	83.49 ± 12.23	84.07 ± 10.10	p = 0.86	r = 0.03	p = 0.86
EPAP (cmH2O)	$EPAP_1$	4.98 (6.01)	5.96 (6.03)	p = 0.06	ρ = 0.25	p = 0.06
	$EPAP_2$	4.99 (5.99)	5.99 (6.00)	p = 0.13	ρ = 0.20	p = 0.13
	$EPAP_3$	4.99 (5.99)	5.99 (5.96)	p = 0.21	ρ = 0.17	p = 0.21
Reached EPAP (% set EPAP)	Reached $EPAP_1$	99.67 (26.90)	99.73 (56.17)	p = 0.42	ρ = 0.10	p = 0.43
	Reached $EPAP_2$	99.75 (25.80)	99.71 (56.89)	p = 0.63	ρ = -0.06	p = 0.64
	Reached $EPAP_3$	99.74 (25.85)	99.73 (41.47)	p = 0.81	ρ = -0.03	p = 0.81
Mean RR (bpm)	Mean RR_1	23.51 (31.75)	21.80 (31.08)	p = 0.58	ρ = -0.07	p = 0.58
	Mean RR_2	24.45 (36.19)	21.17 (31.67)	p = 0.55	ρ = -0.08	p = 0.56
	Mean RR_3	23.83 (36.42)	20.87 (33.20)	p = 0.54	ρ = -0.08	p = 0.54
Reached RR (% back-up frequency)	Reached RR_1	112.81 (115.08)	112.49 (62.35)	p = 0.99	ρ = 0.002	p = 0.99
	Reached RR_2	110.10 (85.61)	111.83 (44.21)	p = 0.60	ρ = 0.07	p = 0.60
	Reached RR_3	111.66 (81.55)	113.06 (42.93)	p = 0.66	ρ = 0.06	p = 0.66
% triggering	$Triggering_1$	47.18 ± 23.59	46.07 ± 21.61	p = 0.86	r = -0.02	p = 0.86
	$Triggering_2$	42.09 ± 24.21	45.86 ± 18.68	p = 0.53	r = 0.08	p = 0.53
	$Triggering_3$	42.77 ± 23.95	46.44 ± 18.59	p = 0.56	r = 0.08	p = 0.56
Leakage (L/min)	$Leakage_1$	37.35 (28.18)	41.71 (72.37)	p = 0.16	ρ = 0.18	p = 0.16
	$Leakage_2$	34.20 (31.81)	36.66 (127.29)	p = 0.27	ρ = 0.14	p = 0.27
	$Leakage_3$	33.39 (43.45)	36.46 (123.97)	p = 0.37	ρ = 0.12	p = 0.37

<u>Supplementary table 5</u>: Comparison of baseline characteristics and BIS parameters between groups and correlation analyses for Mean TcCO2.

1 = value from the night of the TcCO2 and SpO2 measurement; 2 = value from 48h before the overnight measurement; 3 = value from 72 hours before the overnight measurement. ρ = Spearman correlation coefficient; r = Pearson correlation coefficient. Normally distributed data are presented as mean ± standard deviation (SD) and skewed data as median (range). Statistical significance is indicated by an asterisk.

**BIS parameters**		**Comparison between groups**			**Correlation analysis**	
		Normal Mean TcCO2 (n = 59)	Abnormal Mean TcCO2 (n = 10)	p-value	Correlation coefficient	p-value
TV (ml)	TV_1	123.83 (389.56)	72.86 (289.34)	p = 0.25	ρ = -0.15	p = 0.25
	TV_2	115.27 (337.15)	85.75 (278.94)	p = 0.31	ρ = -0.13	p = 0.32
	TV_3	117.56 (331.63)	85.40 (280.06)	p = 0.62	ρ = -0.07	p = 0.62
Reached TV (% target TV)	Reached TV_1	99.77 (190.52)	96.53 (35.86)	p = 0.98	ρ = -0.01	p = 0.97
	Reached TV_2	98.55 (127.18)	96.62 (51.09)	p = 0.63	ρ = -0.08	p = 0.62
	Reached TV_3	100.16 (125.67)	96.29 (52.79)	p = 0.93	ρ = -0.02	p = 0.92
IPAP (cmH2O)	$IPAP_1$	16.77 ± 4.01	16.19 ± 5.64	p = 0.70	*r* = -0.12	p = 0.35
	$IPAP_2$	16.72 ± 3.46	15.83 ± 5.29	p = 0.50	*r* = -0.13	p = 0.33
	$IPAP_3$	16.75 ± 3.57	15.71 ± 5.70	p = 0.47	*r* = -0.15	p = 0.28
Reached IPAP (% set IPAP)	Reached $IPAP_1$	83.13 ± 12.30	85.64 ± 14.60	p = 0.57	*r* = 0.03	p = 0.85
	Reached $IPAP_2$	83.24 ± 11.44	83.61 ± 11.77	p = 0.93	*r* = -0.03	p = 0.81
	Reached $IPAP_3$	83.79 ± 11.45	83.22 ± 11.97	p = 0.89	*r* = 0.06	p = 0.68
EPAP (cmH2O)	$EPAP_1$	5.00 (6.05)	5.50 (3.96)	p = 0.59	ρ = 0.07	p = 0.59
	$EPAP_2$	5.00 (6.00)	5.50 (3.92)	p = 0.76	ρ = 0.04	p = 0.76
	$EPAP_3$	5.01 (5.99)	5.98 (3.92)	p = 0.84	ρ = 0.03	p = 0.84
Reached EPAP (% set EPAP)	Reached $EPAP_1$	99.71 (26.90)	99.61 (56.17)	p = 0.96	ρ = -0.01	p = 0.96
	Reached $EPAP_2$	99.75 (25.90)	99.65 (65.90)	p = 0.33	ρ = -0.13	p = 0.34
	Reached $EPAP_3$	99.75 (25.85)	99.71 (41.47)	p = 0.90	ρ = -0.02	p = 0.90
Mean RR (bpm)	Mean RR_1	23.17 (32.49)	25.23 (30.93)	p = 0.57	ρ = 0.08	p = 0.57
	Mean RR_2	22.43 (37.18)	27.62 (30.97)	p = 0.58	ρ = 0.07	p = 0.58
	Mean RR_3	22.53 (37.26)	25.32 (32.72)	p = 0.85	ρ = 0.03	p = 0.85
Reached RR (% back-up frequency)	Reached RR_1	113.07 (115.08)	111.75 (62.35)	p = 0.71	ρ = -0.05	p = 0.71
	Reached RR_2	110.18 (85.61)	112.02 (44.21)	p = 0.97	ρ = -0.01	p = 0.97
	Reached RR_3	111.81 (81.55)	110.96 (42.93)	p = 0.51	ρ = -0.00	p = 0.52
Triggering (%)	$Triggering_1$	47.90 ± 22.72	41.39 ± 23.43	p = 0.41	*r* = -0.12	p = 0.38
	$Triggering_2$	44.00 ± 22.76	41.21 ± 19.76	p = 0.72	*r* = -0.01	p = 0.97
	$Triggering_3$	44.29 ± 22.61	43.03 ± 20.28	p = 0.88	*r* = -0.01	p = 0.99
Leakage (L/min)	$Leakage_1$	37.73 (72.37)	34.84 (23.75)	p = 0.71	ρ = -0.05	p = 0.71
	$Leakage_2$	36.00 (129.41)	35.18 (21.97)	p = 0.92	ρ = -0.01	p = 0.92
	$Leakage_3$	36.00 (127.26)	40.16 (20.16)	p = 0.82	ρ = 0.03	p = 0.82

Supplementary table 6: Comparison of baseline characteristics and BIS parameters between groups and correlation analyses for Mean SpO2.

1 = value from the night of the TcCO2 and SpO2 measurement; 2 = value from 48h before the overnight measurement; 3 = value from 72 hours before the overnight measurement. ρ = Spearman correlation coefficient; r = Pearson correlation coefficient. Normally distributed data are presented as mean ± standard deviation (SD) and skewed data as median (range). Statistical significance is indicated by an asterisk.

**BIS parameters**		**Comparison between groups**			**Correlation analysis**	
		Normal Mean SpO2 (n = 64)	Abnormal Mean SpO2 (n = 5)	p-value	Correlation coefficient	p-value
TV (ml)	TV_1	122.32 (400.64)	120.28 (238.08)	p = 0.72	ρ = -0.05	p = 0.70
	TV_2	114.87 (355.95)	65.44 (279.34)	p = 0.68	ρ = -0.06	p = 0.66
	TV_3	117.56 (353.05)	65.51 (279.57)	p = 0.76	ρ = -0.05	p = 0.74
Reached TV (% target TV)	Reached TV_1	100.29 (190.52)	73.45 (44.91)	p = 0.23	ρ = -0.19	p = 0.20
	Reached TV_2	98.55 (127.18)	96.93 (22.91)	p = 0.73	ρ = -0.06	p = 0.71
	Reached TV_3	100.27 (125.67)	98.18 (23.00)	p = 0.71	ρ = -0.07	p = 0.68
IPAP (cmH2O)	$IPAP_1$	16.56 ± 4.28	18.22 ± 4.39	p = 0.46	*r* = 0.10	p = 0.46
	$IPAP_2$	16.57 ± 3.86	16.54 ± 3.19	p = 0.98	*r* = -0.003	p = 0.98
	$IPAP_3$	16.60 ± 4.02	16.48 ± 3.37	p = 0.95	*r* = -0.01	p = 0.95
Reached IPAP (% set IPAP)	Reached $IPAP_1$	82.81 ± 12.73	93.85 ± 2.58	p = <0.001*	*r* = 0.22	p = 0.09
	Reached $IPAP_2$	83.30 ± 11.68	82.30 ± 8.50	p = 0.84	*r* = -0.03	p = 0.84
	Reached $IPAP_3$	83.90 ± 11.84	81.60 ± 6.10	p = 0.67	*r* = -0.06	p = 0.67
EPAP (cmH2O)	$EPAP_1$	5.00 (6.05)	6.48 (2.08)	p = 0.20	ρ = 0.17	p = 0.19
	$EPAP_2$	5.00 (6.00)	5.99 (2.30)	p = 0.39	ρ = 0.12	p = 0.37
	$EPAP_3$	5.00 (5.99)	5.99 (2.57)	p = 0.38	ρ = 0.12	p = 0.37
Reached EPAP (% set EPAP)	Reached $EPAP_1$	99.69 (56.16)	99.85 (1.48)	p = 0.17	ρ = 0.19	p = 0.16
	Reached $EPAP_2$	99.75 (56.90)	99.75 (5.30)	p = 0.98	ρ = -0.01	p = 0.97
	Reached $EPAP_3$	99.75 (33.53)	99.72 (10.72)	p = 0.98	ρ = 0.004	p = 0.98
Mean RR (bpm)	Mean RR_1	23.17 (29.96)	31.54 (28.57)	p = 0.62	ρ = 0.07	p = 0.60
	Mean RR_2	22.81 (31.11)	42.57 (32.34)	p = 0.18	ρ = 0.18	p = 0.17
	Mean RR_3	22.53 (31.76)	42.11 (32.25)	p = 0.20	ρ = 0.18	p = 0.19
Reached RR (% back-up frequency)	Reached RR_1	112.66 (115.08)	113.15 (43.13)	p = 0.54	ρ = 0.09	p = 0.52
	Reached RR_2	110.10 (85.61)	121.61 (61.80)	p = 0.17	ρ = 0.18	p = 0.16
	Reached RR_3	111.41 (81.55)	120.31 (60.80)	p = 0.12	ρ = 0.21	p = 0.12
Triggering (%)	$Triggering_1$	46.70 ± 23.53	48.46 ± 6.52	p = 0.71	*r* = 0.02	p = 0.88
	$Triggering_2$	42.57 ± 22.12	54.17 ± 21.99	p = 0.27	*r* = 0.15	p = 0.27
	$Triggering_3$	42.98 ± 22.39	55.32 ± 16.37	p = 0.24	*r* = 0.16	p = 0.24
Leakage (L/min)	$Leakage_1$	37.58 (72.37)	38.08 (15.91)	p = 0.90	ρ = 0.02	p = 0.88
	$Leakage_2$	36.02 (129.41)	35.79 (11.07)	p = 0.74	ρ = -0.05	p = 0.72
	$Leakage_3$	36.10 (127.26)	36.00 (10.49)	p = 0.70	ρ = -0.06	p = 0.68

Supplementary table 6: Correlation analyses for Mean TcCO2 and Mean SpO2.

1 = value from the night of the TcCO2 and SpO2 measurement; 2 = value from 48h before the overnight measurement; 3 = value from 72 hours before the overnight measurement. ρ = Spearman correlation coefficient; r = Pearson correlation coefficient.

**BIS parameters**		**Correlation analysis for Mean TcCO2**		**Correlation analysis for Mean SpO2**	
		Correlation coefficient	p-value	Correlation coefficient	p-value
TV (ml)	TV_1	ρ = -0.26	p = 0.045*	ρ = -0.05	p = 0.71
	TV_2	ρ = -0.19	p = 0.14	ρ = 0.05	p = 0.68
	TV_3	ρ = -0.18	p = 0.18	ρ = 0.12	p = 0.38
Reached TV (% target TV)	Reached TV_1	ρ = -0.18	p = 0.23	ρ = -0.07	p = 0.65
	Reached TV_2	ρ = -0.31	p = 0.04*	ρ = 0.10	p = 0.50
	Reached TV_3	ρ = -0.26	p = 0.10	ρ = 0.18	p = 0.25
IPAP (cmH2O)	$IPAP_1$	*r* = -0.11	p = 0.43	ρ = -0.02	p = 0.89
	$IPAP_2$	*r* = -0.12	p = 0.37	ρ = 0.06	p = 0.64
	$IPAP_3$	*r* = -0.11	p = 0.42	ρ = 0.04	p = 0.78
Reached IPAP (% set IPAP)	Reached $IPAP_1$	*r* = 0.08	p = 0.53	ρ = 0.06	p = 0.67
	Reached $IPAP_2$	*r* = 0.01	p = 0.95	ρ = 0.22	p = 0.09
	Reached $IPAP_3$	*r* = 0.00	p = 1.00	ρ = 0.17	p = 0.22
EPAP (cmH2O)	$EPAP_1$	ρ = 0.16	p = 0.24	ρ = -0.16	p = 0.21
	$EPAP_2$	ρ = 0.19	p = 0.15	ρ = -0.17	p = 0.20
	$EPAP_3$	ρ = 0.17	p = 0.19	ρ = -0.15	p = 0.27
Reached EPAP (% set EPAP)	Reached $EPAP_1$	ρ = -0.01	p = 0.97	ρ = -0.07	p = 0.62
	Reached $EPAP_2$	ρ = -0.18	p = 0.17	ρ = 0.03	p = 0.81
	Reached $EPAP_3$	ρ = -0.06	p = 0.66	ρ = 0.11	p = 0.42
Mean RR (bpm)	Mean RR_1	ρ = -0.02	p = 0.89	ρ = 0.03	p = 0.81
	Mean RR_2	ρ = -0.03	p = 0.80	ρ = -0.03	p = 0.81
	Mean RR_3	ρ = -0.04	p = 0.80	ρ = -0.06	p = 0.66
Reached RR (% back-up frequency)	Reached RR_1	ρ = -0.19	p = 0.14	ρ = -0.12	p = 0.38
	Reached RR_2	ρ = 0.01	p = 0.99	ρ = -0.18	p = 0.16
	Reached RR_3	ρ = -0.02	p = 0.89	ρ = -0.25	p = 0.07
Triggering (%)	$Triggering_1$	*r* = -0.06	p = 0.67	ρ = -0.22	p = 0.10
	$Triggering_2$	*r* = 0.05	p = 0.69	ρ = -0.22	p = 0.09
	$Triggering_3$	*r* = -0.05	p = 0.71	ρ = -0.22	p = 0.11
Leakage (L/min)	$Leakage_1$	ρ = -0.02	p = 0.89	ρ = -0.08	p = 0.55
	$Leakage_2$	ρ = 0.04	p = 0.78	ρ = -0.10	p = 0.44
	$Leakage_3$	ρ = 0.05	p = 0.71	ρ = -0.03	p = 0.86
